# Rescue of Fructose-Induced Metabolic Syndrome by Antibiotics or Faecal Transplantation in a Rat Model of Obesity

**DOI:** 10.1371/journal.pone.0134893

**Published:** 2015-08-05

**Authors:** Blanda Di Luccia, Raffaella Crescenzo, Arianna Mazzoli, Luisa Cigliano, Paola Venditti, Jean-Claude Walser, Alex Widmer, Loredana Baccigalupi, Ezio Ricca, Susanna Iossa

**Affiliations:** 1 Department of Biology, University “Federico II” of Naples, Naples, Italy; 2 Genetic Diversity Centre, ETH Zurich, Zurich, Switzerland; 3 Institute of Integrative Biology (IBZ), ETH Zurich, Zurich, Switzerland; Charité, Campus Benjamin Franklin, GERMANY

## Abstract

A fructose-rich diet can induce metabolic syndrome, a combination of health disorders that increases the risk of diabetes and cardiovascular diseases. Diet is also known to alter the microbial composition of the gut, although it is not clear whether such alteration contributes to the development of metabolic syndrome. The aim of this work was to assess the possible link between the gut microbiota and the development of diet-induced metabolic syndrome in a rat model of obesity. Rats were fed either a standard or high-fructose diet. Groups of fructose-fed rats were treated with either antibiotics or faecal samples from control rats by oral gavage. Body composition, plasma metabolic parameters and markers of tissue oxidative stress were measured in all groups. A 16S DNA-sequencing approach was used to evaluate the bacterial composition of the gut of animals under different diets. The fructose-rich diet induced markers of metabolic syndrome, inflammation and oxidative stress, that were all significantly reduced when the animals were treated with antibiotic or faecal samples. The number of members of two bacterial genera, *Coprococcus* and *Ruminococcus*, was increased by the fructose-rich diet and reduced by both antibiotic and faecal treatments, pointing to a correlation between their abundance and the development of the metabolic syndrome. Our data indicate that in rats fed a fructose-rich diet the development of metabolic syndrome is directly correlated with variations of the gut content of specific bacterial taxa.

## Introduction

It is well-established that a hypercaloric diet, rich in highly refined carbohydrates and fat (Western diet), can induce a wide range of metabolic alterations, including obesity, increased plasma triglyceride concentration, impaired glucose tolerance and insulin resistance [1–3]. A major component of the Western diet is the common monosaccharide fructose, present either free or bound to glucose (i.e. sucrose). Free fructose is found in many fruits and vegetables and is the most abundant component of sweeteners (hydrolyzed corn starch), that are now largely used in western countries. It has been estimated that in the US the load of free fructose has increased from 158.5 kcal per person per day in 1978 to 228 kcal per person per day in 1998 [3]. This substantial increase has paralleled the increased incidence of obesity, leading to the hypothesis that a fructose-rich diet may contribute to the development of obesity and related metabolic disorders [3–5]. However, the contribution of high-fructose diets to the development of obesity remains controversial, since some authors have not observed unequivocal evidence linking fructose consumption with metabolic disorders [2]. To address this issue, studies with animal models have proven particularly informative [6]. A recent study performed with nonhuman primates has shown that even in the absence of weight gain, fructose rapidly causes liver damages and that hepatic steatosis relates to the duration of fructose consumption [7]. Previous experiments showed that, even if the caloric intake is the same, a fructose-rich diet given to adult rats induced obesity (i.e. increased body lipids and increased epidydimal fat) as a consequence of an increased de novo lipogenesis taking place in the liver [8]. In addition, we found that long term consumption of the fructose-rich diet impaired glucose tolerance, induced an oxidative stress status and increased plasma non-esterified-fatty-acids (NEFA) [9], the latter being considered a reliable marker of the development of insulin resistance [10].

In the last decade the rapid development of metagenomic approaches has allowed the analysis of microbial communities present in various districts of the human body. It is now clear that the microbial community of the human gut is complex. It provides a relevant contribution to the metabolic properties of the gastrointestinal tract and by consequence to the health status of the host [11]. The composition of the gut microbiota is influenced by changes in environmental factors, including diet [11–13]. It has been reported that the presence of specific nutrients may have an influence on the composition of the microbial community, leading to changes in the function of the microbiota [14–16].

In this context it is reasonable to assume that a fructose-rich diet could alter the composition of the gut microbiota. However, it is unknown how gut microbiota altered by a high fructose diet would affect overall health. Only very recent studies have started to address the importance of microbiota in animal models or humans exposed to a fructose-rich diet [3]. Hsieh et al [17] reported that the oral administration of a probiotic strain of *Lactobacillus reuteri* improves insulin resistance and reduces hepatic steatosis in rats fed with a fructose-rich diet, suggesting the probiotic-based approach as a promising therapeutic strategy in the treatment of the metabolic syndrome and of the type 2 diabetes.

Here we show that the detrimental effects caused by fructose-induced metabolic syndrome in adult rats were abolished by an antibiotic treatment, suggesting a direct involvement of the microbiota in the induction of the metabolic syndrome. Similar effects, though less evident, were observed when fructose-fed rats were inoculated with faecal samples of rats under standard diet, although they were less evident. A 16S DNA-based analysis of the gut microbiota of the various animals was performed to evaluate the alteration of the macrobiotic gut community composition in animals under different diets.

## Materials and Methods

### Animals and treatments

Male Sprague-Dawley rats (Charles River, Italy), of 100 days of age were used as a model of diet-induced obesity [18]. They were caged singly in a temperature-controlled room (23±1°C) with a 12-h light/dark cycle (06.30–18.30). Treatment, housing, and euthanasia of animals met the guidelines set by the Italian Health Ministry. All experimental procedures involving animals were approved by “Comitato Etico-Scientifico per la Sperimentazione Animale” of the University “Federico II” of Naples.

Rats were divided in two groups, each with the same mean body weight (460±10 g), that were fed either with control diet (Mucedola 4RF21; Settimo Milanese, Milan, Italy) or with a fructose-rich diet, known to induce early signs of obesity within 8 weeks of treatment [8, 9, 19]. The energy content of the two diets was the same, because the composition was the same, except that half of the starch of the control diet had been substituted with fructose in the fructose-rich diet (Table 1). In addition, rats were pair-fed for the whole experimental period, by giving them the same amount of diet, both as weight and as caloric content. Each rat consumed the full portion of the diet fed them each day over the 8 week study period.

**Table 1:** Composition of experimental diets.

	CONTROL DIET	FRUCTOSE DIET
Component (g/100 g)		
Standard chow	100.0	50.5
Sunflower oil		1.5
Casein		9.2
Alphacel		9.8
Fructose		20.4
Water		6.4
AIN-76 mineral mix		1.6
AIN-76 vitamin mix		0.4
Choline		0.1
Methionine		0.1
Gross energy density, kJ/g	17.2	17.2
Metabolisable energy density, kJ/g *	11.1	11.1
Protein, % metabolisable energy	29.0	29.0
Lipids, % metabolisable energy	10.6	10.6
Carbohydrates, % metabolisable energy	60.4	60.4
Of which:		
Fructose	——-	30.0
Starch	45.3	22.8
Sugars	15.1	7.6

*estimated by computation using values (kJ/g) for energy content as follows: protein 16.736, lipid 37.656, and carbohydrate 16.736.

Rats fed the control diet were divided in two groups of six rats each: one group received water supplemented with antibiotic mix (Ampicillin 1 g/L + neomycin 0.5 g/L) (CA rats), while the second group did not receive any further treatment and served as control (C rats). Rats fed the fructose-rich diet were divided in three groups, each composed of six rats: one group received water supplemented with the above antibiotic mix (FA rats), the second group was subjected to microbiota transplantation (FT rats), while the third group did not receive any further treatment (F rats). Placebo gavage was given to all the rats.) The antibiotic mix (Ampicillin + neomycin) was chosen because ampicillin is able to penetrate Gram-positive and some Gram-negative bacteria, while neomycin has excellent activity against Gram-negative bacteria, and has partial activity against Gram-positive bacteria. In addition, both antibiotics are poorly absorbed (or unabsorbed as in the case of neomycin) and thus without any systemic effects [20]. For microbiota transplantation, to ensure that each of the FT rats received a bacterial load similar in number and species, stools from all six control animals were pooled and rats of group FT fed with aliquots of the pooled mixture. To this end, fresh faecal pellets (2 pellets for each rat) from all six donor rats (C rats) were collected and placed in transfer buffer (pre-reduced sterile phosphate buffered saline containing 0.05% cysteine HCl, 2 mL/g) on ice. The faecal pellets were homogenized, centrifuged at 800g for 2 min and the supernatant was collected. Diluted faecal supernatant was then orally inoculated to recipient rats (0.5 mL/rat) every third day during the 8 weeks dietary treatment period. During the treatments, body weight, food and water intake were monitored daily and no adverse effect was observed during the whole experimental period.

### Metabolic analysis

To perform the glucose tolerance test the day before the sacrifice, rats were fasted for 6 hours from 09.00 a.m. A basal, postabsorptive blood sample was obtained from a small tail clip and placed in EDTA—coated tubes and then glucose (2 g/kg body weight) was injected intraperitoneally. Blood samples were collected after 20, 40, 60, 90, 120 and 150 min and placed in EDTA-coated tubes. The blood samples were centrifuged at 1400xg_av_ for 8 min at 4°C. Plasma glucose concentration was measured by a colorimetric enzymatic method (Pokler Italia, Genova, Italy).

Plasma NEFA levels were measured by colorimetric enzymatic method (Roche Diagnostics, Mannheim, Germany). Plasma tumor necrosis factor alpha (TNF-α) concentrations were determined using a rat-specific enzyme linked immunosorbent assay (R&D Systems, MN, USA) according to the manufacturer’s instruction. Plasma lipopolysaccharide (LPS) determinations were performed using a kit based upon a *Limulus amaebocyte* extract (LAL kit; Lonza, Basel, Switzerland).

To evaluate body energy and lipid content, sacrificed rats were killed by decapitation and livers and hindleg skeletal muscles were quickly removed. Guts were cleaned of undigested food and the carcasses were then autoclaved. After dilution into distilled water and subsequent homogenisation of the carcasses, duplicate samples of the homogenised carcass were analyzed for energy content by bomb calorimeter [9]. Total body lipid content was measured by the Folch extraction method [21].

### Isolation of epididymal adipocytes and measurement of in vitro lypolitic capacity

Adipocytes were isolated from intra-abdominal epididymal white adipose tissue (WAT) as previously reported [20]. Aliquots corresponding to 15000 cells were then incubated in the presence of 1 μM isoproterenol, with or without 0.1 μM insulin, for 2 h at 37°C in a shaking bath. At the end of the incubation, aliquots were used for the determination of glycerol production, by incubating samples with Sigma glycerol reagent at 37°C for 15 min and then monitoring absorbance at 540 nm against appropriate standards.

### Preparation of whole tissue homogenates from liver and skeletal muscle

Whole tissue homogenates were prepared from liver and skeletal muscle as previously reported [9, 22]. The extent of the peroxidative processes in whole tissue homogenates was determined by measuring the level of lipid hydroperoxides according to Heath & Tappel [23]. Determination of protein oxidative damage was performed measuring protein-bound carbonyl levels by the procedure of Reznick & Packer [24].

### Western blot quantification of p-Akt in skeletal muscle tissue

Skeletal muscle tissue samples were homogenized in lysis buffer containing 20 mM Tris-HCl (pH 7.5), 150 mM NaCl, 2.7 mM KCl, 5% (v/v) glycerol, 1% (v/v) Triton X-100 and 50 μl/g tissue of protease inhibitor cocktail (all from Sigma-Aldrich Corp., St. Louis, MO, USA) using a Potter homogeniser, shaken for 2 h at 4°C, and centrifuged at 14000 x g for 20 min at 4°C. The supernatants were collected and aliquots were denatured, subjected to electrophoresis and the gels were transferred onto PVDF membranes (Millipore, MA, USA). After preblocking, the membranes were incubated overnight at 4°C with polyclonal antibody for the phosphorylated form of kinase Akt (p-Akt, Cell Signaling, MA, USA, diluted 1:1000 in blocking buffer). After washing, the membranes were incubated 1 hour at room temperature with a anti-rabbit, alkaline phosphatase-conjugated secondary antibody (Promega, WI, USA), and then incubated at room temperature with a chemiluminescent substrate, CDP-Star (Sigma-Aldrich, MO, USA). Data detection was carried out by exposing autoradiography films (Eastman Kodak Company, NY, USA) to the membranes. Quantification of signals was carried out by Un-Scan-It gel software (Silk Scientific, UT, USA). Kinase Akt was detected with polyclonal antibody (Cell Signaling, MA, USA, diluted 1:1000 in blocking buffer) and used to normalize the p-Akt signal.

### Caecal sample preparation, DNA extraction and measurement of caecal glucose and fructose

The ceacal content of 30 rats (six replicates for each of five different fed groups) was squeezed out, collected separately and immediately placed onto dry ice. The total genomic DNA was extracted in triplicate from 200 mg of each caecal microbioma sample using the QIAamp DNA Stool Mini Kit (QIAGEN) following the manufacturer’s instructions.

Caecal content of glucose and fructose was assessed by using colorimetric enzymatic methods (Pokler Italia, Genova, Italy for glucose and Sigma-Aldrich Co., Saint Louis, MO for fructose).

### PCR and amplicon preparation

Primers designed by Caporaso et al. [25] (515F: 5'-GTGCCAGCMGCCGCGGTAA-3') and Wang et al. [26] (939R: 5'-CTTGTGCGGGCCCCCGTCAATTC-3') were used to amplify a ≈ 400nt region of the 16S rRNA gene. The amplicon covers the hyper variable regions V4 and V5. Each 25 μl PCR mix contained 2X Phusion Master Mix (BioLabs Inc., New England), 10 μM of each PAGE-purified primer (Microsynth, Switzerland) and 50 ng of template-DNA. The PCR conditions used were 98°C for 30s, 15 cycles of 98°C for 10s, 52°C for 30s and 72°C for 30s, followed by 72°C for 10min using a SensoQuest PCR cycler (Germany). The PCR products were checked for their length on 1,5% agarose gel with GelRed Nucleic Acid Gel Stain (Biotium Inc., USA) and purified with DNA clean and concentrator-5 kit (Zymo Research, U.S.A). The DNA concentration was determined using Qubit 2.0 Fluorometer and Qubit dsDNA HS Assays Kit (Invitrogen, Life Technologies).

### Illumina library preparation

Index libraries for the purified PCR products were prepared using the TruSeq Nano DNA Sample Prep Kit (HT) following manufacturer’s recommended protocols (Illumina, Inc., San Diego, Ca, USA). The quantity of the different libraries were assessed by qPCR using the Kappa Library Quantification Kit (KAPA Biosystems Inc., USA). Paired-End sequencing (PE-250) was performed on an Illumina MiSeq at the Genomic Diversity Centre (GDC) at the ETH, Zurich, Switzerland following manufacturer’s recommended protocols (Illumina, Inc., San Diego, CA, USA). The MiSeq Control Software Version 2.4 including MiSeq Reporter 2.4 was used for the primary analysis and the de-multiplexing of the raw reads.

### Bioinformatics analyses

Raw reads (reads per run = 1 x10^7^; reads per sample≅ 3 x 10^5^) were quality checked with FastQC (v0.11.2) and overlapping forward and reverse reads were merged and error corrected (minimum overlap 15, maximum overlap 200, and maximum mismatch density 0.25) using FLASh (v.1.2.9,) [27]. Reads that could not be merged were excluded from further analysis. Primer region were identified and trimmed off the ends of the merged reads using Cutadapt (v.1.4) allowing no mismatches. In a subsequent step, the merged and trimmed reads were quality filtered using PrinSeq Lite (v.0.20.4). Reads with mean quality below 25 or any reads containing ambiguous nucleotides (i.e. “N”) were removed. The remaining cleaned reads were de-noised (identity threshold 99%) and chimera checked (de-novo and reference based against the 16S gold reference database provided by QIIME) using Usearch [28] version 6. The reads were binned using reference mapping applying the Usearch option as part of QIIME version 1.6.0 [29]. The remaining reads were passed to the QIIME pipeline. Uclust [23] was used to define Operational Taxonomic Units (OTUs) at 97% sequence identity, which were assigned a taxonomy using the RDP classifier [30]. Representative sequences for each OTU were aligned with PyNast [31] and columns uninformative for phylogeny building were filtered out using Greengenes [32]. The resulting alignments were used to build a phylogeny using FastTree [33]. The principal coordinates analysis (PCoA) was performed on pairwise unweighted UniFrac distances [34]. The sequencing data have been deposited in Sequence Read Archive (SRA), accession number SRP060322.

### Statistical analysis

Data are given as means±SEM of six different rats. Statistical analyses were performed by one-way analysis of variance followed by Tukey post hoc test. Probability values less than 0.05 were considered significant. All analyses were performed using GraphPad Prism 4 (GraphPad Software, San Diego, CA, USA). The Analysis of Similarity (ANOSIM) was performed by QIIME and used to assess the difference in OTU composition between samples collected after different treatments. ANOSIM provides an R statistics, which can range from -1 to 1 (albeit values < 0 are rarely obtained). R values > 0.75 indicate well separated microbial compositions; R > 0.5 overlapping, but clearly different communities; and R < 0.25 practically not separable communities. The significance of ANOSIM test was assessed using a randomization procedure whereby the value R was recomputed 999 times.

## Results

### Antibiotics or faecal samples do not affect the fructose-induced increase in body energy and lipid content

**Fig 1:**
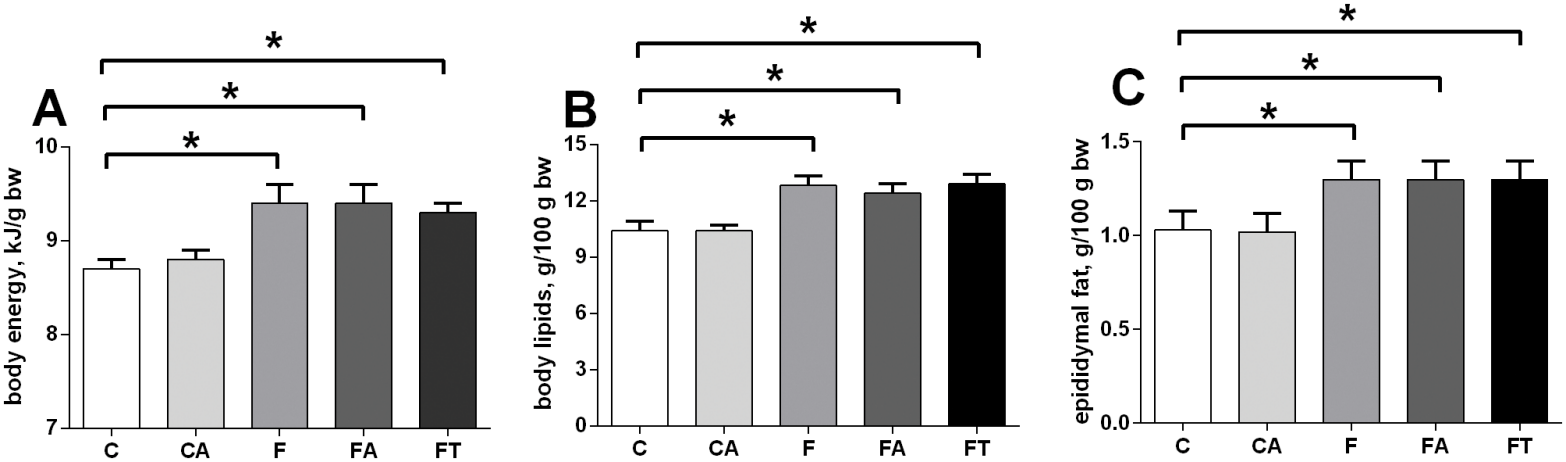
Antibiotics or faecal samples do not affect the fructose-induced increase in body energy and lipid content. Animal whole body composition was obtained by measurement of body energy (panel A), lipids (panel B) and epididymal fat (C) content in control (C), control+antibiotic (CA), fructose-fed (F), fructose-fed+antibiotic (FA) and fructose-fed+faecal samples (FT) rats. Values are reported as means±SEM of six different rats. * P< .05 (one-way ANOVA followed by Tukey post-test).

A first metabolic characterization was carried out by analyzing the animal whole body composition. As shown in Fig 1, fructose-fed rats displayed significantly higher body energy (panel A), lipids (panel B) and epididymal fat compared to controls. The treatment with antibiotics or faecal samples did not affect the increase in body energy (Fig 1A) or lipids (Fig 1B and 1C) due to the diet. No significant difference was found in final body weight (C = 538±20; CA = 540±15; F = 541±15; FA = 537±17; FT = 540±23 g) and in body weight gain (C = 78±5; CA = 80±5; F = 81±8; FA = 77±7; FT = 80±3 g) between the five groups of rats. Since changes in energy intake are the primary drive of obesity development, metabolisable energy (ME) intake was monitored throughout the experimental period to verify whether the fructose-induced increase in body energy and lipid content was due to an increase of ME. To this aim, food intake and energy loss through faeces and urines were analyzed (Methods) and indicated that ME intake was similar in all experimental groups (C = 19,700±1,200 kJ, CA = 19,400±1,050 kJ, F = 19,500±1,100 kJ, FA = 19,200±990 kJ, FT = 19,500±1,000 kJ). In addition, the caecal content of glucose and fructose was measured to assess whether dietary treatment could modify substrate availability in the hindgut, but no significant variation en caecal glusose and fructose was found between the five groups of rats (S1 Table).

### Antibiotics or faecal samples reduce markers of the fructose-induced metabolic syndrome

**Fig 2:**
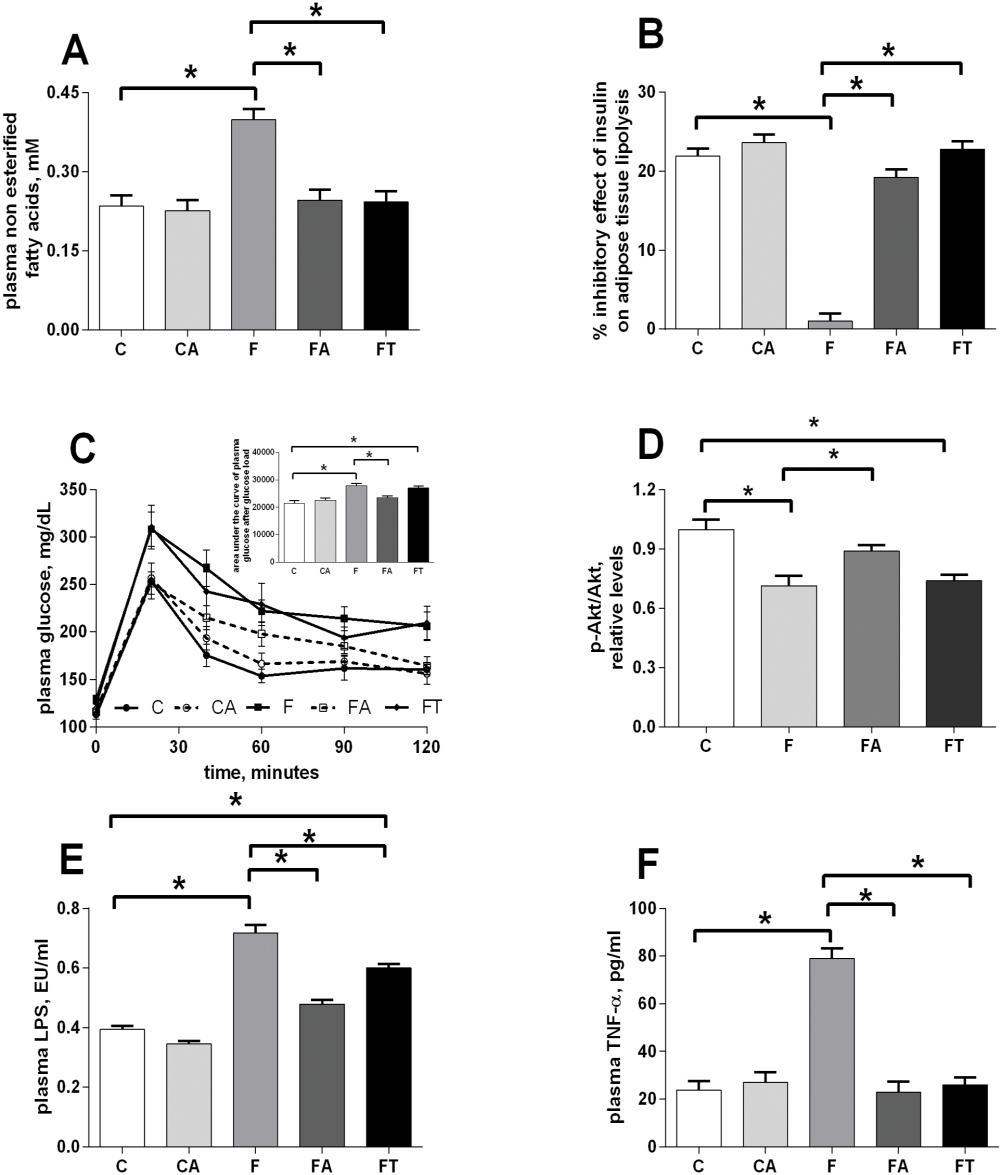
Antibiotics or faecal samples reduce markers of the fructose-induced metabolic syndrome. Plasma NEFA (A) and inhibitory effect of insulin on lipolysis in epididymal WAT (B) were assessed as first markers of metabolic syndrome. Changes in plasma glucose after the administration of a given dose of glucose were plotted over the time (C) and the area under the curves was calculated and used to estimate glucose tolerance (inset in panel C), while the contribution of skeletal muscle to changes in glucose tolerance was estimated by measuring the degree of phosphorylation of the kinase Akt, a distal effector of insulin signalling in this tissue (D). As a third marker of the development of a metabolic syndrome, the plasma concentration of LPS (E) and TNF-alpha (F) were considered. Values obtained in control (C), control+antibiotic (CA), fructose-fed (F), fructose-fed+antibiotic (FA) and fructose-fed+faecal samples (FT) rats are reported as means±SEM of six different rats. * P< .05 (one-way ANOVA followed by Tukey post-test). EU = endotoxin unit.

The levels of plasma NEFA and glucose intolerance were followed as early markers of the development of metabolic derangement and insulin resistance. Fig 2A shows that the significant increase in plasma NEFA found in fructose-fed rats (group F vs C) was almost completely reversed by treatment with antibiotics (group FA) or faecal samples (group FT). In addition, the inhibitory effect of insulin on lipolysis in epididymal WAT was nearly absent in fructose-fed rats (group F), but was completely restored by treatment with antibiotics (group FA) or faecal samples (group FT) (Fig 2B).

Changes in plasma glucose after the administration of a given dose of glucose were plotted over the time and the area under the curves was calculated and used to estimate glucose tolerance. The significantly higher values observed in fructose-fed rats (group F vs C) indicate that, with the same glucose injection, the increase in plasma glucose levels was more marked in fructose-fed rats (Fig 2C, inset). This reduced glucose tolerance was abolished by antibiotic treatment (group FA), while the treatment with faecal samples only slightly increased glucose tolerance (Fig 2C). To assess the contribution of skeletal muscle to changes in glucose tolerance, we investigated a distal effector of insulin signalling in this tissue, and we found that pAkt levels were significantly lower in fructose-fed (group F) and fructose-fed+faecal samples (group FT) rats, while this decrease was abolished by antibiotic treatment (group FA) (Fig 2D and S1 Fig).

As markers of obesity-induced inflammation, the plasma concentration of LPS and TNF-alpha were considered, since metabolic endotoxaemia is associated with obesity, metabolic syndrome and type 2 diabetes [35]. A statistically significant increase in plasma LPS levels was measured in fructose-fed rats and was partly reduced by both treatments, with antibiotic and faecal samples (Fig 2E). In addition, plasma concentrations of TNF-alpha were found four-fold higher in fructose-fed rats than in control animals (Fig 2F) and both antibiotic treatment and faecal transplant were able to completely abolish this increase (Fig 2F).

### Antibiotics or faecal samples reduce the fructose-induced tissue oxidative stress

**Fig 3:**
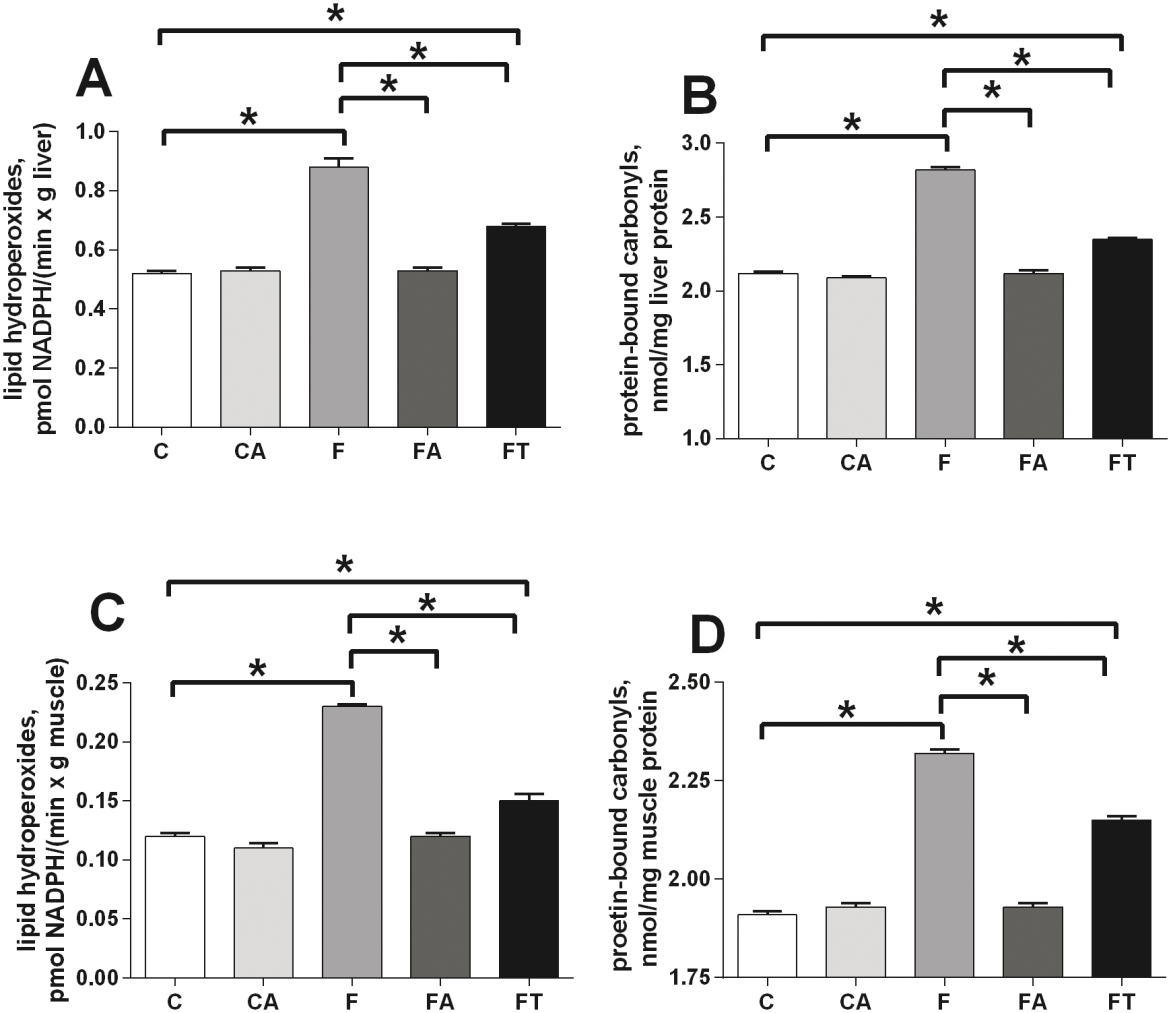
Antibiotics or faecal samples reduce the fructose-induced tissue oxidative stress. Oxidative damage in liver (A, B) and skeletal muscle (C, D) was evaluated by assessing the levels of damaged lipids (A, C) and proteins (B, D) in control (C), control+antibiotic (CA), fructose-fed (F), fructose-fed+antibiotic (FA) and fructose-fed+faecal samples (FT) rats. Values are reported as means±SEM of six different rats. * P< .05 (one-way ANOVA followed by Tukey post-test).

Oxidative damage in liver and skeletal muscle was evaluated by assessing the levels of damaged lipids (panels A and C of Fig 3) and proteins (panels B and D of Fig 3) in both tissues. Fructose-fed rats showed higher levels of oxidative stress than control rats in liver (panels A and B of Fig 3) and skeletal muscle (panels C and D of Fig 3). Treatments with antibiotics or faecal samples totally or partially reversed the effect in both tissues, respectively (Fig 3).

### Gut microbial composition

In order to investigate the effect of the fructose-rich diet on the gut microbial composition and the efficacy of the faecal transplant in abolishing such effects we used a 16S DNA-sequencing approach. As detailed below, the analysis on samples for rats of the control group was in agreement with previous data for rats under standard diet regimen with *Firmicutes* much more abundant than *Bacteroidetes*, *Proteobacteria* and *Tenericutes* [36].

**Fig 4:**
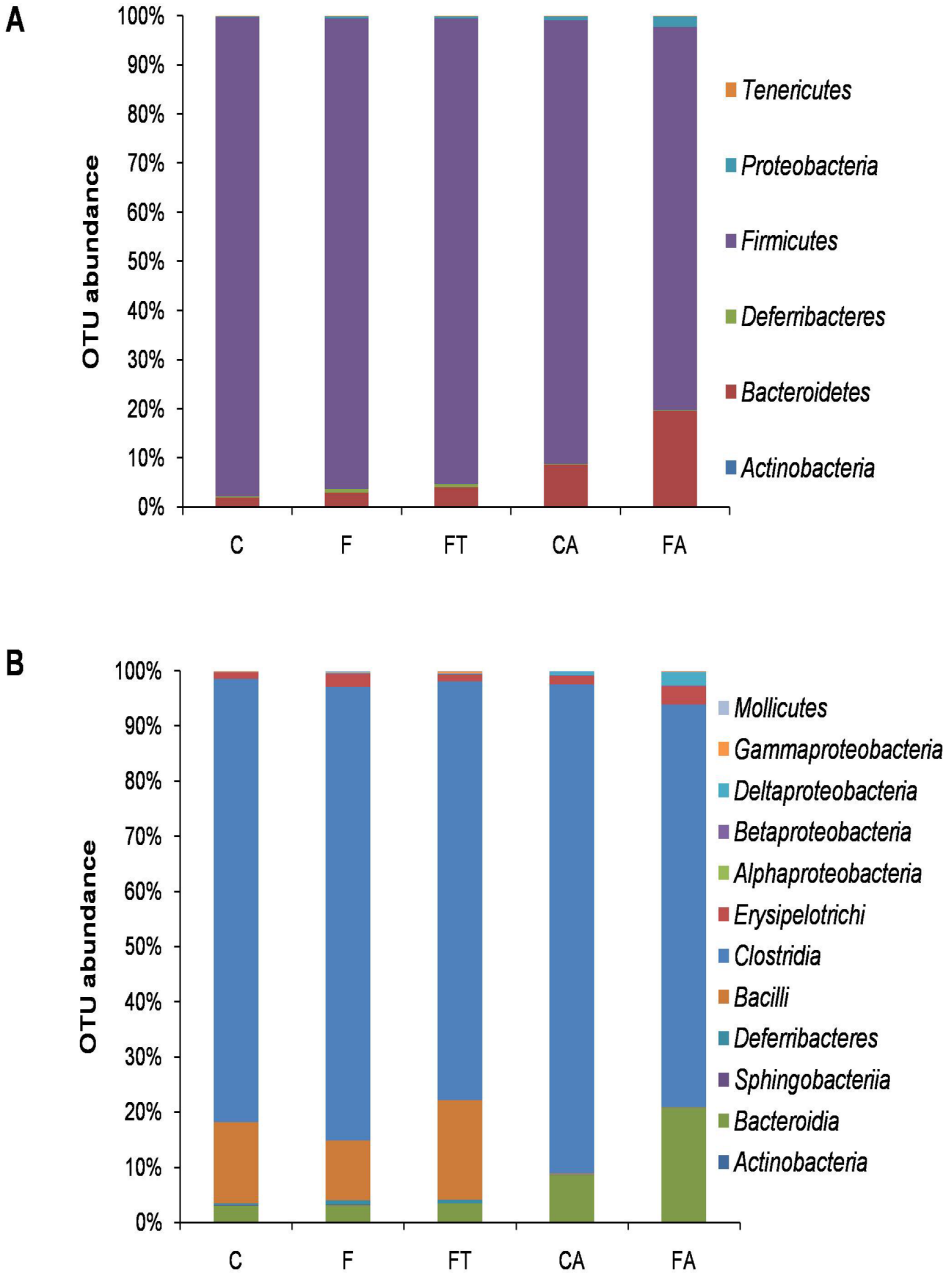
Relative Operational Taxonomic Units (OTUs) abundance at the *Phylum* (A) or Class (B) level in control (C), control+antibiotic (CA), fructose-fed (F), fructose-fed+antibiotic (FA) and fructose-fed+faecal samples (FT) rats. The bar plot shows how the caecal microbiota distribution of each sample changes in rats of the various groups.

The identified phylotypes show that *Firmicutes* were the most abundant bacteria in all five diet groups (97–77%) while *Bacteroidetes*, *Proteobacteria* and *Tenericutes* were always less represented (19–2%, 2–0.5% and 0.2–0.07%, respectively) (Fig 4A). A significant increase in the number of *Bacteroidetes* was observed in the gut of animals treated with antibiotics (Fig 4A). At the class level, *Clostridia* are the most abundant organisms in all five groups, followed by *Bacilli* in groups C, F and FT and by *Bacteroidia* in CA and FA (Fig 4B). Independently from the diet regimen, *Bacilli* were almost totally absent in the gut of animals treated with antibiotics (Fig 4B).

**Fig 5:**
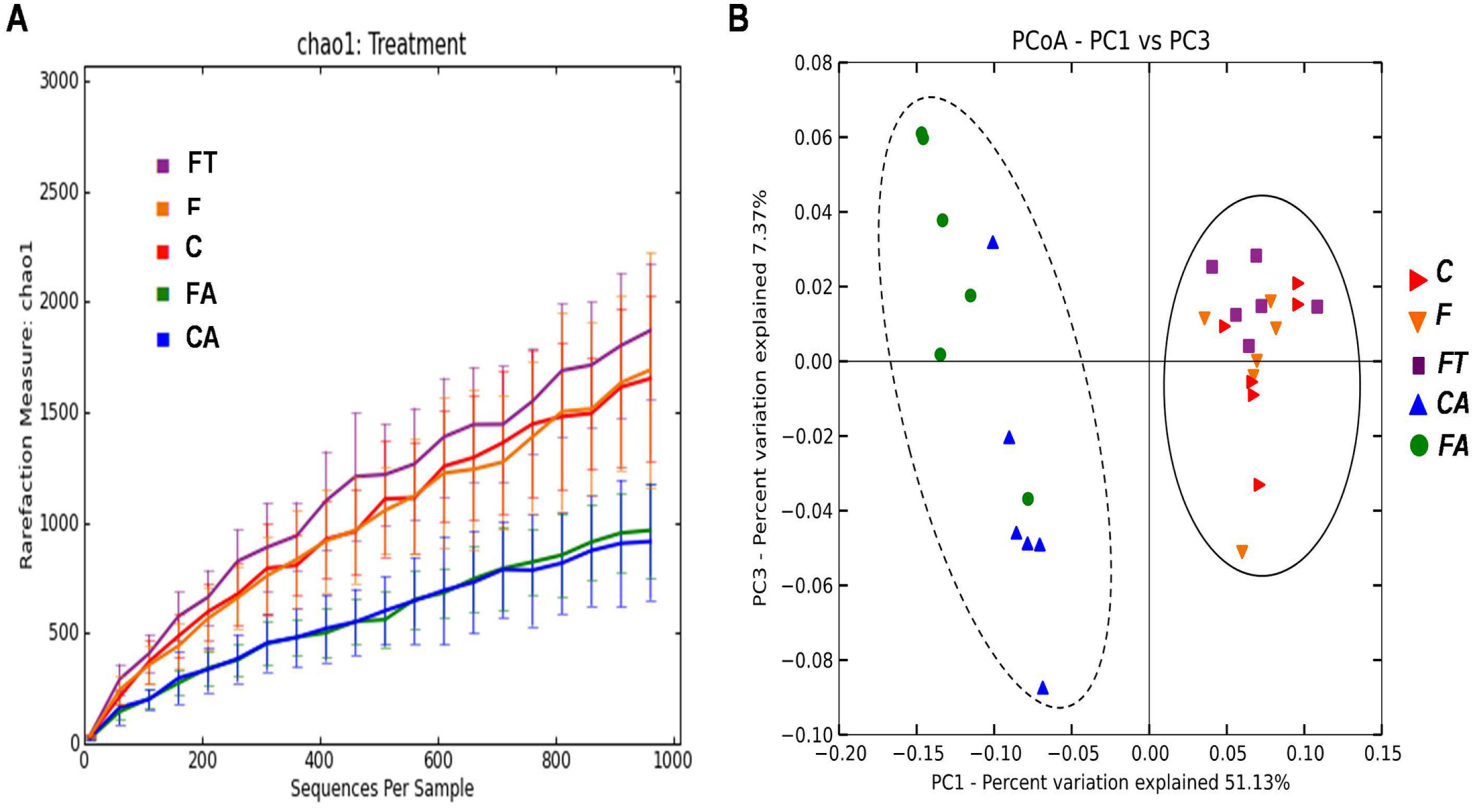
Variation of bacterial composition in/among the samples in control (C), control+antibiotic (CA), fructose-fed (F), fructose-fed+antibiotic (FA) and fructose-fed+faecal samples (FT) rats. (A) Alpha-diversity plot. The Chao1 species richness estimator indicates a higher microbial complexity in the C, F, FT groups than in the CA and FA groups. Values are reported as means±SEM of six different rats. (B) Beta-diversity is shown by Principal Coordinates Analysis (PCoA), based on UniFrac method. The plot displays two main clusters: C, F and FT belong to cluster I (straight line), while CA and FA belong to cluster II (dashed line).

The bacterial content of the samples was estimated by Chao1 algorithm (alpha-diversity metric) and showed a decreased microbial complexity in samples CA and FA compared to the other three groups (Fig 5A). This is not surprising and indicates that the antibiotic treatment had strongly altered the gut microbial composition causing a reduction of the microbial diversity. In order to analyze relationships among samples based on differences in phylogenetic diversity (beta-diversity metric), principle coordinates (PC) were calculated using UniFrac distances [34] between samples. Sample distribution in the PCoA plot (Fig 5B) clearly showed two main clusters, one containing CA and FA samples and another one containing C, F and FT samples. Similar results were obtained when the PCoA plot was rerun exclusively with C, F and FT rats (S2 Fig). Results of Fig 5, in agreement with the taxonomical assignment at *phylum* and class level of Fig 4 indicate that the microbial composition of the caecum of animals treated with antibiotics completely differs from that of animals not under antibiotic treatment. Because of the strong alterations, only data from animals not treated with antibiotics (C, F and FT groups) were considered for further analysis. The detailed composition of microbiota from CA and FA rats is reported in S3 Fig.

**Fig 6:**
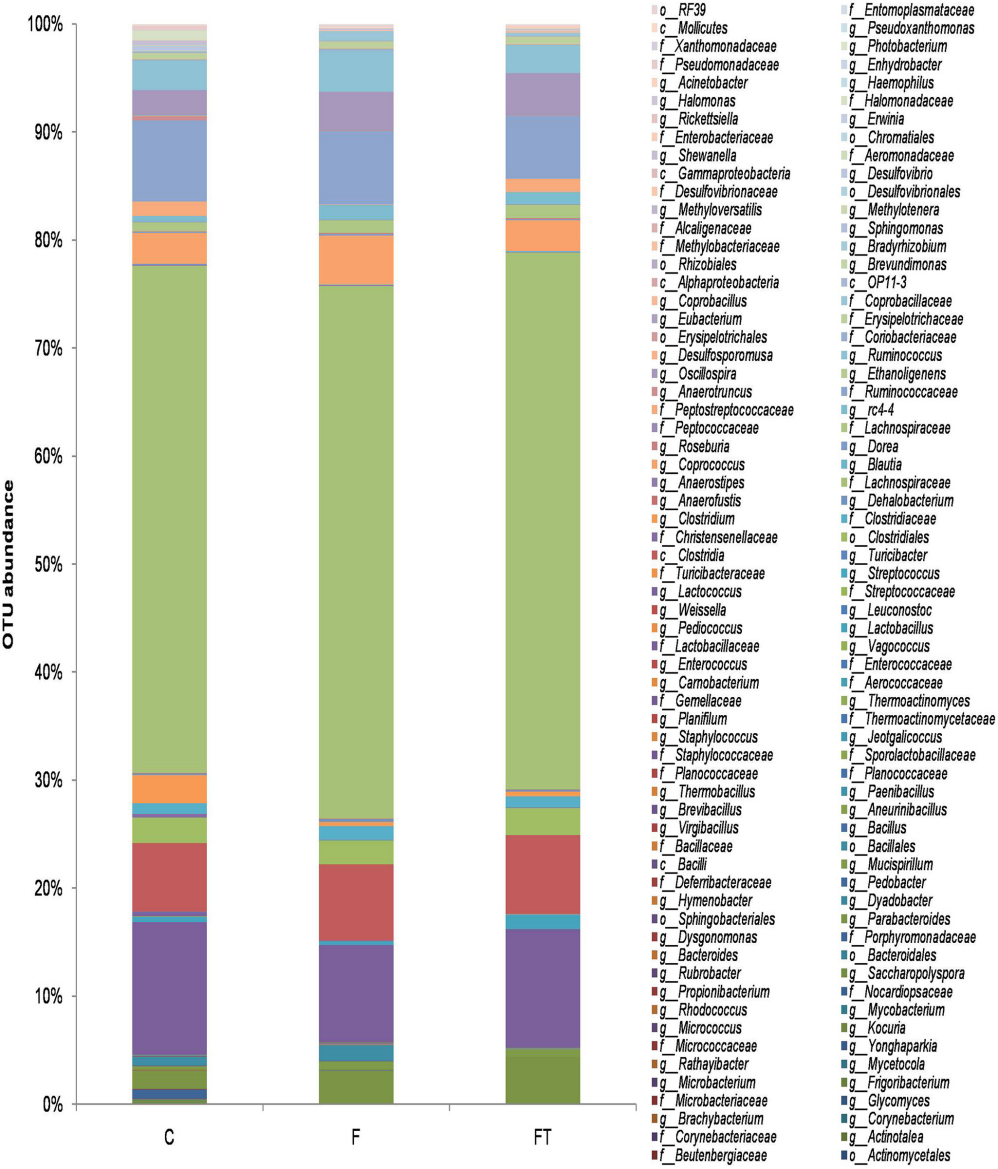
Relative Operational Taxonomic Units (OTUs) abundance at the *genus* level in control (C), fructose-fed (F), and fructose-fed+faecal samples (FT) rats. Composition of caecal microbiota of rats from different diet groups as revealed by Illumina sequencing of V4-V5 hypervariable region of 16S rRNA gene. Population analyses for each diet group show phylotypes at genus level (when it was possible) and are reported as means of six rats for each diet group (*global* R = 0.71 *p < 0*.*001*, Analysis of Similarity).

The overall microbial composition of the gut of rats of groups C, F and FT at the genus level were altered by the different diets and treatments (global R = 0.71 p < 0.001). The most abundant genera (mostly belonging to the *Clostridia* class) appeared similarly represented in the gut of all three groups (Fig 6).

A more detailed analysis of groups C and F showed that the representativeness of eight genera and of the *Coriobacteriaceae* family (in bold in Table 2) was significantly different (P < .05) and suggested an effect of the fructose-rich diet on those bacteria.

**Table 2:** Bacteria differentially distributed in the caecum of control and fructose-fed rats.

Phylum	Class	Order	Family	Genus	*C group* *	*F group* *	*P valueC versus F*
*Bacteroidetes*	*Bacteroidia*	*Bacteroidales*	*Bacteroidaceae*	***Bacteroides***	0.0310±0.01	0.0040±0.004	**0.0499567**
*Firmicutes*	*Bacilli*	*Bacillales*	*Staphylococcaceae*		0.0390±0.01	0.0260±0.005	0.379299
				Staphylococcus	0.0150±0.005	0.0040±0.002	0.113753
		*Lactobacillales*	*Enterococcaceae*	Enterococcus	0.0300±0.003	0.0960±0.03	0.077031
			*Lactobacillaceae*		13.0820±3.6	8.4010±2.4	0.069455
				***Lactobacillus***	0.6050±0.03	0.3640±0.07	**0.0352972**
	*Clostridia*	*Clostridiales*	*Clostridiaceae*		1.0710±0.03	1.1860±0.013	0.524552
				***Clostridium***	2.7640±0.9	0.3590±0.07	**0.0462856**
			*Dehalobacteriaceae*	*Dehalobacterium*	0.1810±0.03	0.2640±0.01	0.0635174
			*Lachnospiraceae*		49.978±1.7	45.7570±2.1	0.0544198
				*Anaerostipes*	0.1580±0.02	0.1090±0.02	0.182093
				*Blautia*	0.0150±0.005	0.0530±0.02	0.122038
				***Coprococcus***	3.0780±0.1	4.1950±0.2	**0.0042314**
				*Dorea*	0.1260±0.02	0.1530±0.03	0.502034
				***Roseburia***	0.0180±0.005	0.0700±0.02	**0.0411975**
			*Peptococcacaee*	***rc4-4***	0.6130±0.06	1.3010±0.1	**0.0028319**
			*Peptostreptococcaceae*		1.4240±0.8	0.0180±0.01	0.130464
			*Ruminococcacae*		7.9680±0.7	6.3220±1.1	0.23389
				*Oscillospira*	2.5100±0.3	3.2970±0.3	0.137306
				***Ruminococcus***	2.9680±0.02	3.5280±0.09	**0.0007972**
		*Coriobacteriales*	***Coriobacteriaceae***		0.0450±0.02	0.1580±0.01	**0.0051196**
	*Erysipelotrichi*	*Erysipelotrichales*	*Erysipelotrichaceae*		0.6320±0.6	0.6500±0.5	0.968611
				*Eubacterium*	0.0320±0.02	0.0090±0.009	0.106268
			*Coprobacillaceae*		0.1990±0.07	0.8520±0.7	0.126056
				***Coprobacillus***	0.0290±0.01	0.0830±0.01	**0.0007489**
*Tenericutes*	*Mollicutes*	*RF39*			0.1050±0.07	0.2610±0.1	0.144464

* Percentage of Operational Taxonomic Units abundance in the sample. The values are reported as means ± SEM of six different rats.

P< .05 values are reported in bold and indicate a significant difference between control (C) and fructose-fed (F) rats.

**Fig 7:**
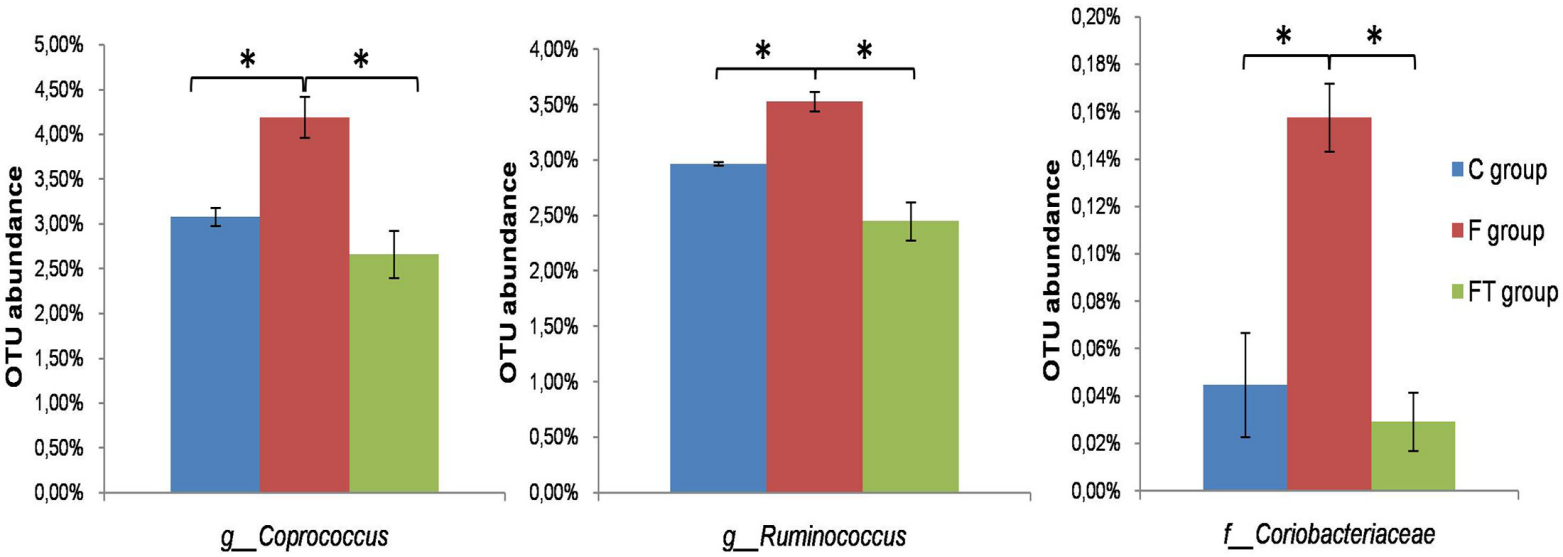
Taxonomical units restored by faecal transplantation. The histograms show the restoration of two genera and one family distributions after faecal trasplantation. Their increase, due to the fructose-rich diet, is rescued by the faecal treatment. Values are reported as means±SEM of six different rats. * P< .05 (one-way ANOVA followed by Tukey post-test). C = control, F = fructose-fed, FT = fructose-fed+faecal samples.

For these eight genera and one family we then analyzed data of the FT group and observed that the representativeness of two genera, *Coprococcus* and *Ruminococcus*, and of the *Coriobacteriaceae* family was restored to levels similar to those observed in the C group (P < .05) (Fig 7). These results suggest a direct correlation between the number of members of the *Coprococcus* and *Ruminococcus* genera and of the *Coriobacteriaceae* family present in the gut and the development of the metabolic syndrome.

For this reason we also analyzed the presence of these three bacterial groups in rats of the CA and FA groups, that do not have signs of metabolic syndrome. In both antibiotic-treated groups the representativeness of the *Coprococcus* and *Ruminococcus* genera was lower than in the F group (Table 3), thus supporting a direct correlation between their representativeness and the development of the metabolic syndrome. For the *Coriobacteriaceae* family such correlation was, instead, not supported by the analysis of the antibiotic-treated groups (Table 3).

**Table 3:** Representativeness of bacterial groups rescued by faecal transplant and antibiotic treatment.

Family	Genus	Group				
		C	F	FT	CA	FA
*Lachnospiraceae*	*Coprococcus*	3.078 ± 0.1	4.195 ± 0.2*	2.600 ± 0.3**	1.100 ± 0.3**	0.500 ± 0.08**
*Ruminococcacae*	*Ruminococcus*	2.968 ± 0.02	3.528 ± 0.09*	2.480 ± 0.2**	3.000± 0.1**	2.700 ± 0.2**
*Coriobacteriaceae*		0.045 ± 0.02	0.158 ± 0.01*	0.029 ± 0.01**	0.610 ± 0.05**	0.420 ± 0.12**

The values are expressed as percentage of Operational Taxonomic Units abundance and are reported as means ± SEM of six different rats. C = control, F = fructose-fed, FT = fructose-fed+faecal samples, CA = control+antibiotic, FA = fructose-fed+antibiotic. rats. (*p< .05 compared to C rats, ** P < .05 compared to F rats, one-way ANOVA followed by Tukey post-test).

## Discussion

The main result of this study is that in fructose-fed rats the development of metabolic syndrome directly correlates with the alteration of the microbial composition of the gut. Markers of metabolic syndrome, increased in fructose-fed rats, are reversed by treatment with an antibiotic mixture and partly by faecal transplant of rats under standard diet.

It has been recently reported that microbiota transplantation through surgical extraction of donor caecal content and administration to the recipient rats by oral gavage was able to reshape indigenous gut microbial community to an extent not previously anticipated [36]. Here we show that a similar reshaping can be obtained without the need of surgical extraction of caecal bacteria, but simply using faecal content, and, more importantly, that the reshaping of gut microbiota occurs in concomitance with metabolic improvement in obese rats.

The animal model used in the present study is represented by adult rats that became obese after long-term feeding with a fructose-rich diet [8, 9, 20]. Fructose-fed obese rats also display increased plasma NEFA, a reliable marker of the development of insulin resistance [10], but this metabolic alteration is fully reversed by treatment with antibiotic or faecal samples. In addition, the inhibition of lipolysis by insulin in WAT is almost completely lost in fructose-fed rats, but treatment with antibiotic or faecal samples is able to restore insulin sensitivity at the level of control rats.

The loss of insulin sensitivity in WAT can be driven by inflammation [37], and here we show that fructose-fed rats exhibited higher plasma LPS and TNF-α, that could therefore contribute to derangements in WAT function. Treatment with antibiotic or faecal samples in fructose-fed rats greatly reduced plasma LPS and completely abolishes the increase in plasma TNF-α, and these changes could be at the basis of the restored insulin sensitivity in WAT in these rats. The increased plasma LPS in fructose-fed rats and its reversal by antibiotic treatment is in agreement with previous findings obtained in mice made obese by high fat or high-fructose diet [38–40]. The increased gut permeability that can be deduced from increased plasma LPS has been documented also in fructose-fed mice through a decrease in the expression of the proteins of the tight junctions, probably arising from the cellular stress imposed to enterocytes by the uncontrolled metabolism of fructose by fructokinase [41]. On the other hand, we cannot exclude that administration of faecal extracts was only marginally reversing the effect of fructose diet on plasma LPS, because it caused an increase in total intestinal LPS. However, to our knowledge, our study is the first to show partial reversal of systemic inflammation in response to treatment with faecal transplantation.

The glucose intolerance found in fructose-fed rats was abolished by treatment with antibiotic but not by faecal transplant. The lack of effect of faecal transplant on glucose tolerance in the present study is at variance with results obtained in human studies [42], but it should be taken into account that in the cited experiment the transplant was carried out by duodenal gavage, whereas in the present study we have used oral gavage. Skeletal muscle greatly contributes to glucose disposal under the control of insulin [43], so we investigated a distal effector of insulin signalling in skeletal muscle, the kinase Akt. The degree of Akt activation was reduced by fructose-rich diet but significantly increased in fructose-fed rats treated with antibiotic. Since LPS directly induces insulin resistance in skeletal muscle [44], and antibiotic treatment completely abolished the diet-induced increase in plasma LPS, we can hypothesize that LPS could contribute to the development of glucose intolerance induced by high fructose diet.

Our present results highlight an increased oxidative damage to lipid and proteins in liver and skeletal muscle of fructose-fed rats, which is fully prevented by antibiotic treatment, and only partly reversed by faecal transplant. This pattern of variation can be explained by modulation of plasma LPS by diet and treatments, since LPS has a well known pro-oxidant effect [45].

The reduction of all markers of metabolic syndrome, inflammation and tissue oxidative stress in fructose-fed animals under antibiotic treatment clearly indicates that the metabolic disorders are somehow due to the fructose-induced alteration of the gut microbiota. On the other hand, the lack of any effect of antibiotic treatment on metabolic parameters in rats fed a standard diet suggests that gut microbiota exerts a negative impact on the metabolic phenotype of the rat only in fructose-induced pathologic conditions. As expected, the gut microbial composition of rats under antibiotic treatment strongly differed from that of animals not treated with antibiotics and was characterized by a reduced diversity.

The fructose-rich diet altered the microbial composition of the gut with eight bacterial genera and a family that significantly differed between rats of groups C and F. The lack of difference in caecal content of glucose and fructose in the five different grouos of rats let us to hypothesize that the effect of fructose-rich is diet is not mediated by changes in substrate availability in the hindgut.

For two of those genera, *Coprococcus* and *Ruminococcus*, and for members of the *Coriobacteriaeae* family, the representativeness was rescued by the faecal treatment and, therefore, directly correlated with the reduction of markers of metabolic syndrome, inflammation and tissue oxidative stress. Consistently, the representativeness of the two bacterial genera was also very low in the antibiotic-treated rats, that did not show signs of metabolic alterations. Those results point to a member of the *Coprococcus* and *Ruminococcus* genera as directly involved in the fructose-driven metabolic syndrome.

*Coprococcus* and *Ruminococcus* are two genera of the Lachnospiraceae family of Clostridia respectively grouping three and thirteen species. These two genera have been included in the list of about 160 bacterial groups dominating the human gastro-intestinal tract [46] and have never been associated to diseases or gut inflammations before. We report here, for the first time, a correlation between the relative abundance of members of the *Coprococcus* and *Ruminococcus* genera and markers of metabolic syndrome.

Metabolic and inflammation markers induced by the fructose-rich diet were completely rescued by the antibiotic treatment and only partially rescued by the faecal transplant. At least two alternative working hypotheses can explain the different responses to the two treatments. A first possible explanation is that the antibiotic treatment reduced or totally eliminated bacteria relevant for the development of the metabolic syndrome, present in animals of the F group and in part also in the FT group. An alternative possibility is suggested by data of Table 2, indicating that bacteria of the *Coprococcus* genus were more drastically affected by the antibiotic treatment (from 4.19% to 0.5% of relative OTU abundance between F and FA groups) than by the faecal transplant (from 4.19% to 2.6% of relative OTU abundance between F and FT), and therefore pointing to the relative abundance of members of the *Coprococcus* genus in animals under a fructose-rich diet as important for the development of the metabolic syndrome. Further experiments will be needed to solve this point that represents a challenging future task.

## Supporting Information

S1 Checklist The ARRIVE Guidelines Checklist. (PDF)

S1 Fig Representative western blots of kinase Akt (Akt), a distal effector of insulin signalling, and its degree of phosphorylation on Ser^473^ (p-Akt) in skeletal muscle samples from control, fructose-fed, fructose-fed+antibiotic and fructose-fed+faecal samples rats. Numbers 1 to 6 indicate six different samples from six different rats. Densitometric analysis of the p-Akt and Akt blots was carried out and the results are shown in Fig 3.
(TIF)

S2 Fig Variation of bacterial composition in/among the samples in control (C), fructose-fed (F), and fructose-fed+faecal samples (FT) rats. Beta-diversity is shown by Principal Coordinates Analysis (PCoA), based on UniFrac method.
(TIF)

S3 Fig Relative Operational Taxonomic Units (OTUs) abundance at the *genus* level in control+antibiotic (CA) and fructose+antibiotic (FA) rats. Composition of caecal microbiota of CA and FA rats as revealed by Illumina sequencing of V4-V5 hypervariable region of 16S rRNA gene. Population analyses for each diet group show phylotypes at genus level (when it was possible) and are reported as means of six rats for each diet group.
(TIF)

S1 Table Caecal content of glucose and fructose in control, fructose-fed, fructose-fed+antibiotic and fructose-fed+faecal samples rats. Values are reported as means±SEM of six different rats. C = control, F = fructose-fed, CA = control+antibiotic, FA = fructose-fed+antibiotic, FT = fructose-fed+faecal samples.
(DOCX)

## References

[1] Johnson RJ, Segal MS, Sautin Y, Nakagawa T, Feig DI, Kang DH, et al Potential role of sugar (fructose) in the epidemic of hypertension, obesity and the metabolic syndrome, diabetes, kidney disease, and cardiovascular disease. Am J Clin Nutr. 2007;86: 899–906. 17921363 10.1093/ajcn/86.4.899

[2] Tappy L, Le KA, Tran C, Paquot N. Fructose and metabolic diseases: New findings, new questions, Nutrition 2010;26: 1044–1049. 10.1016/j.nut.2010.02.014 20471804

[3] Payne AN, Chassard C, Lacroix C. Gut microbial adaptation to dietary consumption of fructose, artificial sweeteners and sugar alcohols: implications for host—microbe interactions contributing to obesity. Obesity 2012;13: 799–809. 10.1111/j.1467-789X.2012.01009.x 22686435

[4] Bray GA, Nielsen SJ, Popkin BM. Consumption of high-fructose corn syrup in beverages may play a role in the epidemic of obesity. Am J Clin Nutr. 2004;79: 537–543. 15051594 10.1093/ajcn/79.4.537

[5] Marriot BP, Cole N, Lee E. National Estimates of Dietary Fructose Intake Increased from 1977 to 2004 in the United States. J. Nutr. 2009; 139: 1228S–1235S. 10.3945/jn.108.098277 19403716

[6] Li S, Zhang HJ, Hu CC, Lawrence F, Gallagher KE, Surapaneni A, et al Assessment of diet-induced obese rats as an obesity model by comparative functional genomics. Obesity 2008;16: 811–818. 10.1038/oby.2007.116 18239588

[7] Kavanagh K, Wylie AT, Tucker KL, Hamp TJ, Gharaibeth RZ, Cullen JM. Dietary fructose induces endotoxemia and hepatic injury in calorically controlled primates. Am J Clin Nutr. 2013;98: 349–357. 10.3945/ajcn.112.057331 23783298 PMC3712547

[8] Crescenzo R, Bianco F, Falcone I, Coppola P, Liverini G, Iossa S. Increased hepatic de novo lipogenesis and mitochondrial efficiency in a model of obesity induced by diets rich in fructose. Eur J Nutr. 2013;52: 537–545. 10.1007/s00394-012-0356-y 22543624

[9] Crescenzo R, Bianco F, Coppola P, Mazzoli A., Cigliano L., Liverini G. et al Increased skeletal muscle mitochondrial efficiency in rats with fructose-induced alteration in glucose tolerance. Br J Nutr. 2013;110:1996–2003. 10.1017/S0007114513001566 23693085

[10] Karpe F, Dickmann JR, Frayn KN. Fatty Acids, Obesity, and Insulin Resistance: Time for a Reevaluation. Diabetes 2011;60: 2441–2449. 10.2337/db11-0425 21948998 PMC3178283

[11] McNulty NP, Wu M, Erickson AR, Pan C, Erickson BK, Martens EC, et al Effects of diet on resource utilization by a model human gut microbiota containing bacteroides cellulosilyticus WH2, a symbiont with an extensive glycobiome. PLoS Biol. 2013;11: e1001637 10.1371/journal.pbio.1001637 23976882 PMC3747994

[12] David LA, Maurice CF, Carmody RN, Gootenberg DB, Button JE, Wolfe BE, Turnbaugh PJ et al Diet rapidly and reproducibly alters the human gut microbiome. Nature 2014; 505: 559–563. 10.1038/nature12820 24336217 PMC3957428

[13] Daniel H, Moghaddas Gholami A, Berry D, Desmarchelier C, Hahne H, Loh G, et al High-fat diet alters gut microbiota physiology in mice. ISME J. 2014; 8: 295–308. 10.1038/ismej.2013.155 24030595 PMC3906816

[14] Tilg H, Moschen AR, Kaser A. Obesity and the microbiota. Gastroenterol. 2009;136: 1476–1483. 10.1053/j.gastro.2009.03.030 19327360

[15] Faith JJ, McNulty NP, Rey FE, Gordon JI. Predicting a human gut microbiota's response to diet in gnotobiotic mice. Science 2011;333: 101–104. 10.1126/science.1206025 21596954 PMC3303606

[16] Walker AW, Ince J, Duncan SH, Webster LM, Holtrop G, Ze X, et al Dominant and diet-responsive groups of bacteria within the human colonic microbiota. ISME J. 2011;5: 220–230. 10.1038/ismej.2010.118 20686513 PMC3105703

[17] Hsieh FC, Lee CL, Chai CY, Chen WT, Lu YC, Wu CS. Oral administration of Lactobacillus reuteri GMNL-263 improves insulin resistance and ameliorates hepatic steatosis in high fructose-fed rats. Nutr Metab. 2013;10: 35 10.1186/1743-7075-10-35 PMC3637306 23590862

[18] Buettner R, Scho¨lmerich J, Bollheime LC. High-fat diets: modeling the metabolic disorders of human obesity in rodents. Obesity 2007;15: 798–808. 17426312 10.1038/oby.2007.608

[19] Crescenzo R, Bianco F, Coppola P, Mazzoli A, Valiante S., Liverini G, et al Adipose tissue remodeling in rats exhibiting fructose-induced obesity. Eur J Nutr. 2014;53: 413–419. 10.1007/s00394-013-0538-2 23728711

[20] Ferrier L, Berard F, Debrauwer L, Chabo C, Langella P, Bueno L, et al Impairment of the intestinal barrier by ethanol involves enteric microflora and mast cell activation in rodents. Am J Pathol. 2006;168: 1148–1154. 16565490 10.2353/ajpath.2006.050617 PMC1606551

[21] Folch J, Lees M, Stanley GHS A. simple method for the isolation and purification of total lipids from animal tissues. J Biol Chem. 1957;226: 497–510. 13428781

[22] Crescenzo R, Bianco F, Falcone I, Prisco M, Liverini G, Iossa S. Alterations in hepatic mitochondrial compartment in a model of obesity and insulin resistance. Obesity 2008;16: 958–964. 10.1038/oby.2008.10 18277391

[23] Heath RL, Tappel AL. A new sensitive assay for the measurement of hydroperoxides. Anal Biochem. 1976;76: 184–191. 998964 10.1016/0003-2697(76)90277-3

[24] Reznick AZ, Packer L. Oxidative damage to proteins: spectrophotometric method for carbonyl assay. Meth Enzymol. 1994;233: 357–363. 8015470 10.1016/s0076-6879(94)33041-7

[25] Caporaso JG, Lauber CL, Walters WA, Berg-Lyons D, Lozupone CA, Turnbaugh PJ, et al Global patterns of 16S rRNA diversity at a depth of millions of sequences per sample. Proc Natl Acad Sci. U S A 2010;108: 4516–4522. 10.1073/pnas.1000080107 20534432 PMC3063599

[26] Wang Y, Qian PY. Conservative fragments in bacterial 16S rRNA genes and primer design for 16S ribosomal DNA amplicons in metagenomic studies. PLoS One 2009;4(10):e7401 10.1371/journal.pone.0007401 19816594 PMC2754607

[27] Magoc T, Salzberg S. FLASH: Fast length adjustment of short reads to improve genome assemblies. Bioinformatics 2011; 27: 2957–2963. 10.1093/bioinformatics/btr507 21903629 PMC3198573

[28] Caporaso JG, Kuczynski J, Stombaugh J, Bittinger K, Bushman FD, Costello EK, et al QIIME allows analysis of high-throughput community sequencing data. Nat Meth. 2010;7(5): 335–366. 10.1038/nmeth.f.303 PMC3156573 20383131

[29] Edgar RC. Search and clustering orders of magnitude faster than BLAST. Bioinformatics 2010;26(19): 2460–2461. 10.1093/bioinformatics/btq461 20709691

[30] Wang Q, Garrity GM, Tiedje JM, Cole JR. Naive Bayesian classifier for rapid assignment of rRNA sequences into the new bacterial taxonomy. Appl Environ Microbiol. 2007;73(16): 5261–5267. 17586664 10.1128/AEM.00062-07 PMC1950982

[31] Caporaso JG, Bittinger K, Bushman FD, DeSantis TZ, Andersen GL, Knight R. PyNAST: a flexible tool for aligning sequences to a template alignment. Bioinformatics 2010;26(2): 266–267. 10.1093/bioinformatics/btp636 19914921 PMC2804299

[32] DeSantis TZ, Hugenholtz P, Larsen N, Rojas M, Brodie EL, Keller K, et al Greengenes, a chimera-checked 16S rRNA gene database and workbench compatible with ARB. Appl Environ Microbiol. 2006;72(7): 5069–5072. 16820507 10.1128/AEM.03006-05 PMC1489311

[33] Price MN, Dehal PS, Arkin AP. FastTree 2—approximately maximum-likelihood trees for large alignments. PLoS One 2010;5(3): e9490 10.1371/journal.pone.0009490 20224823 PMC2835736

[34] Lozupone C, Knight R. UniFrac: a new phylogenetic method for comparing microbial communities. Appl Environ Microbiol. 2005;71(12): 8228–8235. 16332807 10.1128/AEM.71.12.8228-8235.2005 PMC1317376

[35] Piya MK, Harte AL, McTernan PG. Metabolic endotoxaemia: is it more than just a gut feeling? Curr Opin Lipidol. 2013;24: 78–85. 10.1097/MOL.0b013e32835b4431 23298961

[36] Manichanh C, Reeder J, Gibert P, Varela E, Llopis M, Antolin M, et al Reshaping the gut microbiome with bacterial transplantation and antibiotic intake. Genome Res. 2010;20: 1411–1419 10.1101/gr.107987.110 20736229 PMC2945190

[37] van de Woestijne P, Monajemi H, Kalkhoven E, Visseren FLJ. Adipose tissue dysfunction and hypertriglyceridemia: mechanisms and management. Obes Rev. 2011;12: 829–840. 10.1111/j.1467-789X.2011.00900.x 21749607

[38] Bergheim I, Weber S, Vos M, Kramer S, Volynets V, Kaserouni S, et al Antibiotics protect against fructose-induced hepatic lipid accumulation in mice: role of endotoxin. J Hepatol. 2008;48: 983–992. 10.1016/j.jhep.2008.01.035 18395289

[39] Membrez M, Blancher F, Jaquet M, Bibiloni R, Cani PD, Burcelin RG, et al Gut microbiota modulation with norfloxacin and ampicillin enhances glucose tolerance in mice. FASEB J. 2008;22: 2416–2426. 10.1096/fj.07-102723 18326786

[40] Carvalho BM, Guadagnini D, Tsukumo DML, Schenka AA, Latuf-Filho P, Vassallo J, et al Modulation of gut microbiota by antibiotics improves insulin signaling in high-fat fed mice. Diabetologia 2012;55: 2823–2834. 10.1007/s00125-012-2648-4 22828956

[41] Johnson RJ, Rivard C, Lanaspa MA, Otabachian-Smith S, Ishimoto T, Cicerchi C, et al Fructokinase, Fructans, Intestinal Permeability, and Metabolic Syndrome: An Equine Connection? J Equine Vet Sci. 2013;33: 120–126. 23439477 10.1016/j.jevs.2012.05.004 PMC3576823

[42] Vrieze A, Van Nood E, Holleman F, Salojärvi J, Kootte RS, Bartelsman JFWM, et al Transfer of intestinal microbiota from lean donors increases insulin sensitivity in individuals with metabolic syndrome. Gastroenterol. 2012;143: 913–916. 10.1053/j.gastro.2012.06.031 22728514

[43] Stump CS, Henriksen EJ, Wei Y, Sowers JR. The metabolic syndrome: role of skeletal muscle metabolism. Ann Med. 2006;38: 389–402. 17008303 10.1080/07853890600888413

[44] Liang H, Hussey SE, Sanchez-Avila A, Tantiwong P, Musi N. Effect of lipopolysaccharide on inflammation and insulin action in human muscle. PLoS One 2013;8(5): e63983 10.1371/journal.pone.0063983 23704966 PMC3660322

[45] Nishio K, Horie M, Akazawa Y, Shichiri M, Iwahashi H, Hagihara Y, et al Attenuation of lipopolysaccharide (LPS)-induced cytotoxicity by tocopherols and tocotrienols. Redox Biol. 2013;1(1): 97–103. 24024142 10.1016/j.redox.2012.10.002 PMC3757666

[46] Hakansson A. and Molin G. Gut Microbiota and Inflammation. Nutrients 2011;3:637–682. 10.3390/nu3060637 22254115 PMC3257638

