# Supplementary figures and images for: Rescue of Fructose-Induced Metabolic Syndrome by Antibiotics or Faecal Transplantation in a Rat Model of Obesity
Source: PLoS One. 2015 Aug 5;10(8):e0134893. doi: 10.1371/journal.pone.0134893 (PMC4526532; doi:10.1371/journal.pone.0134893)

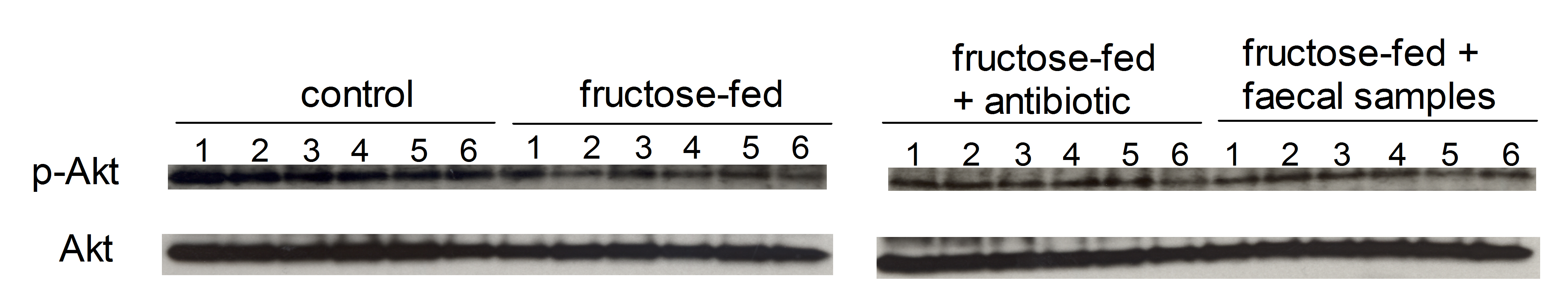

Supplement: S1 Fig — Numbers 1 to 6 indicate six different samples from six different rats. Densitometric analysis of the p-Akt and Akt blots was carried out and the results are shown in Fig 3. (TIF) [file pone.0134893.s002.tif]

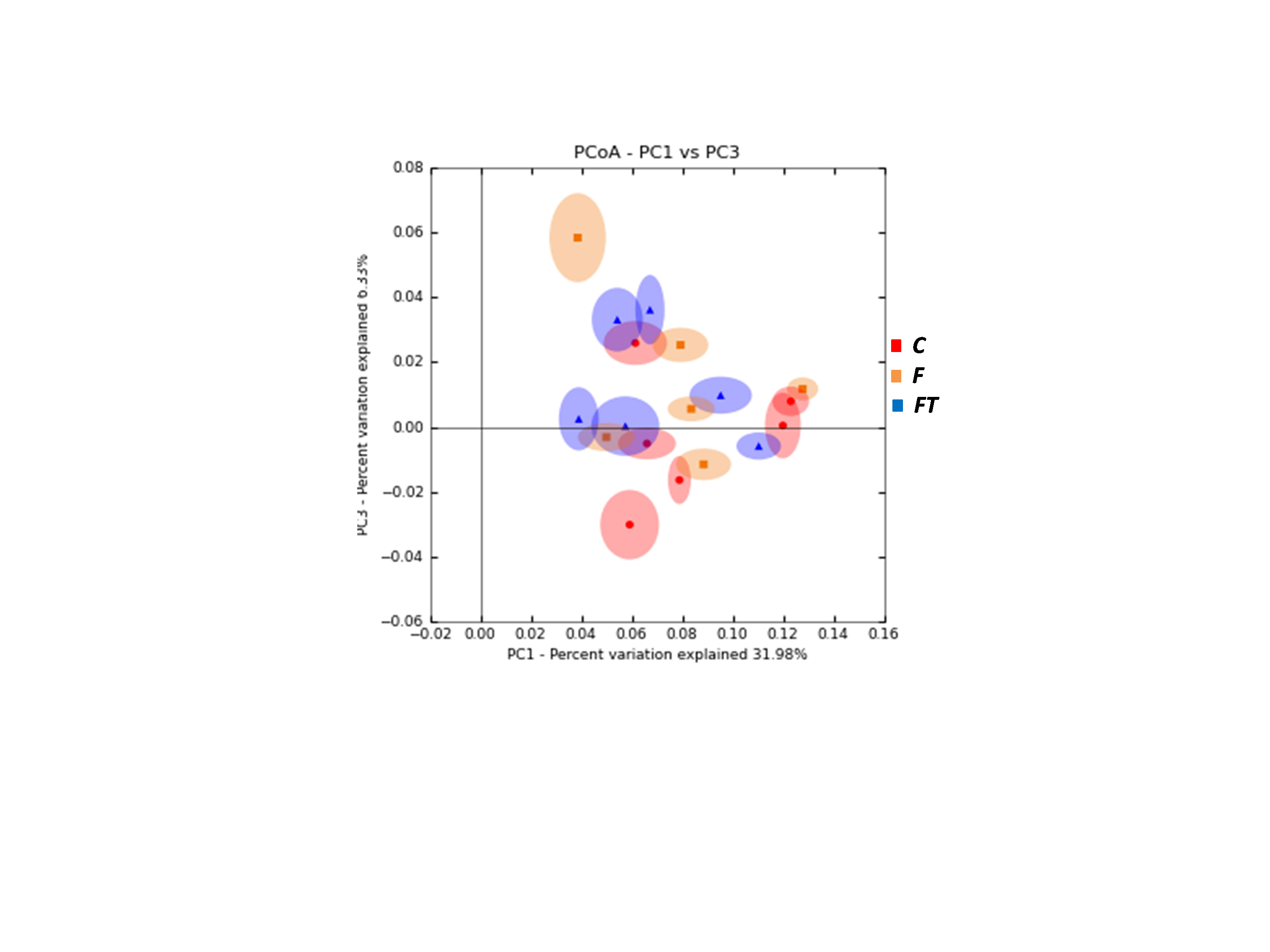

Supplement: S2 Fig — Beta-diversity is shown by Principal Coordinates Analysis (PCoA), based on UniFrac method. (TIF) [file pone.0134893.s003.tif]

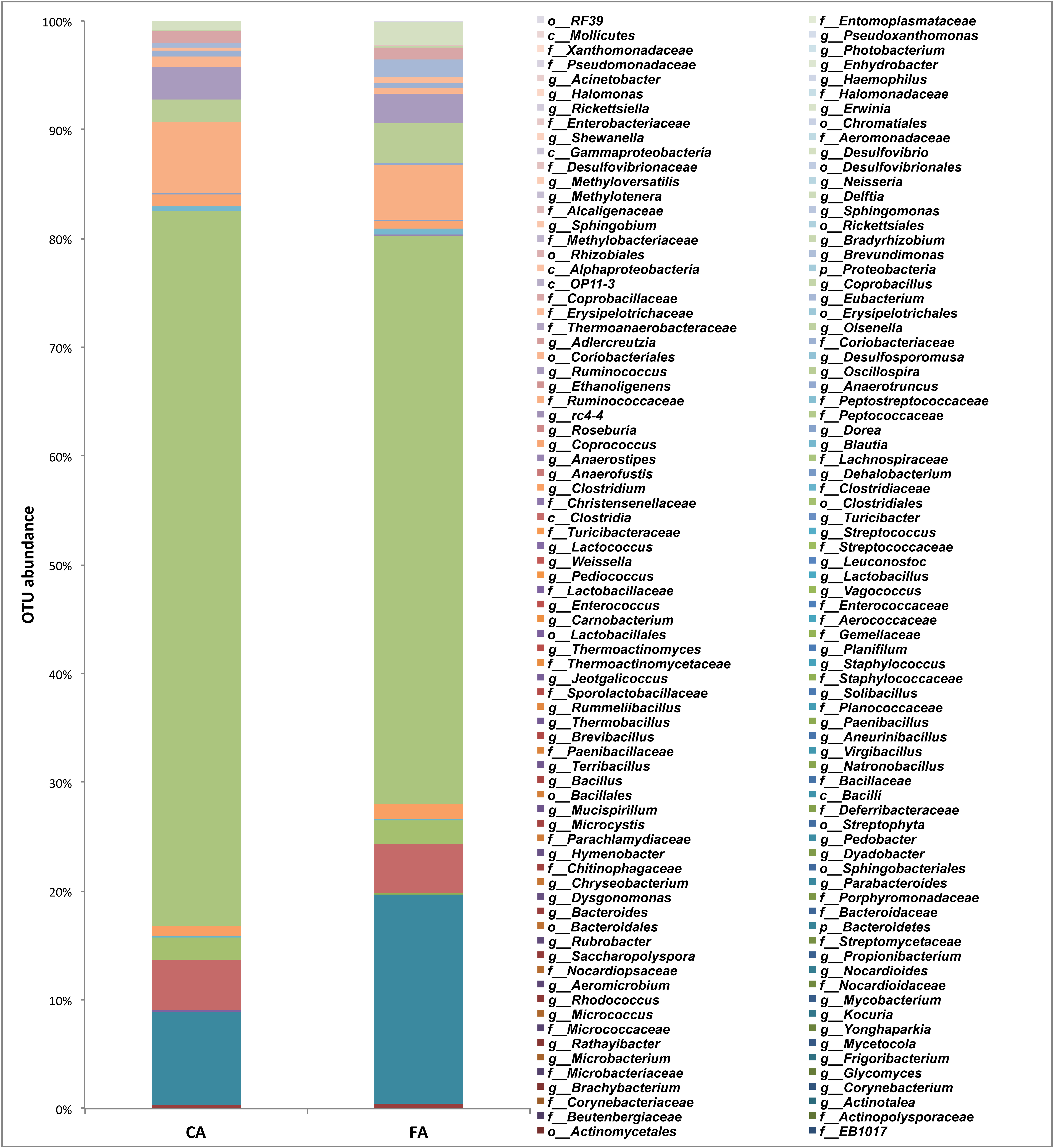

Supplement: S3 Fig — Composition of caecal microbiota of CA and FA rats as revealed by Illumina sequencing of V4-V5 hypervariable region of 16S rRNA gene. Population analyses for each diet group show phylotypes at genus level (when it was possible) and are reported as means of six rats for each diet group. (TIF) [file pone.0134893.s004.tif]
