# Supplementary material for: Rescue of Fructose-Induced Metabolic Syndrome by Antibiotics or Faecal Transplantation in a Rat Model of Obesity
Source: PLoS One. 2015 Aug 5;10(8):e0134893. doi: 10.1371/journal.pone.0134893 (PMC4526532; doi:10.1371/journal.pone.0134893)
Supplement: S1 Table — Values are reported as means±SEM of six different rats. C = control, F = fructose-fed, CA = control+antibiotic, FA = fructose-fed+antibiotic, FT = fructose-fed+faecal samples. (DOCX) [file pone.0134893.s005.docx]

**Table S1**

|  | **C** | **F** | **CA** | **FA** | **FT** |
| --- | --- | --- | --- | --- | --- |
| Caecal glucose content, mg/ml | 1.43±0.05 | 1.52±0.04 | 1.34±0.07 | 1.50±0.03 | 1.47±0.03 |
| Caecal fructose content, mg/ml | 1.92±0.16 | 1.80±0.15 | 1.63±0.21 | 1.48±0.13 | 1.48±0.13 |
